# Formation of a Key Intermediate Complex Species in Catalytic Hydrolysis of NH_3_BH_3_ by Bimetal Clusters: Metal-Dihydride and Boron-Multihydroxy

**DOI:** 10.3389/fchem.2020.00604

**Published:** 2020-09-11

**Authors:** W. J. Yan, Y. F. Zheng, T. W. Zhou, G. Z. Wang, D. D. Wang, H. K. Yuan

**Affiliations:** ^1^School of Physical Science and Technology, Southwest University, Chongqing, China; ^2^School of Mechatronics and Information Engineering, Chongqing College of Humanities, Science & Technology, Chongqing, China; ^3^School of Electronic Information Engineering, Yangtze Normal University, Chongqing, China

**Keywords:** DMol^3^, first-principal calculation, bimetal clusters, catalytic, DFT

## Abstract

The hydrolysis of AB (AB, NH_3_BH_3_) with the help of transition metal catalysts has been identified as one of the promising strategies for the dehydrogenation in numerous experiments. Although great progress has been achieved in experiments, evaluation of the B-N bond cleavage channel as well as the hydrogen transfer channel has not been performed to gain a deep understanding of the kinetic route. Based on the density functional theory (DFT) calculation, we presented a clear mechanistic study on the hydrolytic reaction of AB by choosing the smallest NiCu cluster as a catalyst model. Two attacking types of water molecules were considered for the hydrolytic reaction of AB: stepwise and simultaneous adsorption on the catalyst. The Ni and Cu metal atoms play the distinctive roles in catalytic activity, i.e., Ni atom takes reactions for the H_2_O decomposition with the formation of [OH]^−^ group whereas Cu atom takes reactions for the hydride transfer with the formation of metal-dihydride complex. The formation of Cu-dihydride and B-multihydroxy complex is the prerequisite for the effectively hydrolytic dehydrogenation of AB. By analyzing the maximum barrier height of the pathways which determines the kinetic rates, we found that the hydride hydrogen transferring rather than the N-B bond breaking is responsible to the experimentally measured activation energy barrier.

## 1. Introduction

Among many practical hydrogen storage materials, ammonia-borane (AB, NH_3_BH_3_) is believed to be an attractive solid hydrogen storage candidate owing to its high hydrogen content, nontoxic, excellent solubility, and stability in water (Peng and Chen, 2008; Hamilton et al., 2009; Staubitz et al., 2010; Jiang and Xu, 2011; Sanyal et al., 2011; Thorne et al., 2011; Lu and Xu, 2012; Xi et al., 2012). To enhance the kinetics of the hydrogen release for its on-board applications, intensive efforts have been made in solid state by thermolysis dehydrogenation (Demirci and Miele, 2009; Hamilton et al., 2009) and in solution by hydrolysis and methanolysis dehydrogenation (Xu and Chandra, 2007; Umegaki et al., 2009; Chua et al., 2011). In the absence of suitable catalysts, the thermal/hydrolytic decomposition to generate the hydrogen is a very slow process and it has to undergo several step reactions at very high temperature (Hu et al., 1978; Matus et al., 2007; Stowe et al., 2007; Huang and Autrey, 2012), which seriously restricts the development of AB as a promising hydrogen storage material. In the perspective of hydrolysis dehydrogenation, finding high active and low-cost catalysts to make AB completely release its hydrogen under moderate conditions is a key point for this system.

Up to now, there has been a steady growth in a number of publications dealing with the catalytic hydrolysis of AB to generate the hydrogen at low temperature (Jiang and Xu, 2011; Patel and Miotello, 2015). Originally, noble metal catalysts such as Au (Chandra and Xu, 2007), Pt (Chandra and Xu, 2006a, 2007; Mohajeri et al., 2007; Chen et al., 2014), Pd (Chandra and Xu, 2007; Metin et al., 2011; Kılıç et al., 2012), Rh (Chandra and Xu, 2006a, 2007; Zahmakiran and Özkar, 2009; Karahan et al., 2012), Ru (Chandra and Xu, 2007; Rachiero et al., 2011a; Can and Metin, 2012; Liang et al., 2012; Zhou Q. et al., 2016), and Ir (Wang et al., 2017b) were extensively explored, although their expensive costs are the dominate disadvantage for the potential applicability. Along with the development of cheaper catalysts without the loss of highly efficient, nanoparticle based on more abundant first-row transition metals Fe (Xu and Chandra, 2006; Yan J. M. et al., 2008; Dinç et al., 2012), Co (Xu and Chandra, 2006; Demirci and Miele, 2010; Yan et al., 2010b; Metin and Özkar, 2011; Li et al., 2017), Ni (Xu and Chandra, 2006; Kalidindi et al., 2008a; Yan et al., 2009; Metin et al., 2010; Metin and Özkar, 2011; Li et al., 2017; Wang et al., 2017a), and Cu (Xu and Chandra, 2006; Kalidindi et al., 2008a,b; Zahmakiran et al., 2010) have also been tentatively studied, however, they have only moderate catalysis and lack the desiring stability in the hydrolysis conditions. To get over the challenges of cost expensiveness of the noble metal as well as the catalytic limitation of transition metal, the modulation of the noble metal content by alloying with first-row transition metal was considered as a feasible solution, because their active sites could be maintained and their active capabilities could be improved in alloying catalysts. As one expected, most bimetallic catalysts such as Cu(Fe, Ni, Ru) (Rachiero et al., 2011b; Lu et al., 2013, 2014; Zhang et al., 2016), (Pd, Au, Ru)Co (Yan et al., 2010a; Chen et al., 2011; Sun et al., 2011; Lu et al., 2012; Wang et al., 2012), (Au, Ag, Pt, Ru)Ni (Chen et al., 2007, 2012; Yao C. F. et al., 2008; Jiang et al., 2010), and (Ni, Cu)Pd (Çiftci and Metin, 2014; Güngörmez and Metin, 2015) have been reported to exhibit the better performance than the monometal catalysts for the hydrogen generation from the hydrolysis of AB. It was deduced that the formations of the heterometallic bonds can adjust the bonding (molecular) orbitals of catalyst surface atoms to reactant with AB and H_2_O molecules. This can stabilize the reaction intermediates and lead to the improvement of catalytic activity on AB for hydrogen generation (Lu et al., 2013). The behind reason is attributed to the synergistic effect between the heterometallic atoms that originates from the electronic effect (such as the adjustment of *d*-band level or charge transfers) and the structural morphology (such as the formation of core-shell or mixing structures).

By measuring the composition of hydrolytic products in the presence of various transition metal-based catalysts (Yao C. F. et al., 2008; Rachiero et al., 2011b; Chou et al., 2012; Liu et al., 2012; Komova et al., 2016), it was confirmed that 1 mole of AB is hydrolyzed to give nearly 1 mole of ${\text{NH}}_{4}^{+}$ ions and 3 moles of H_2_ gas. For example, Yao C. F. et al. (2008) have chemically identified that the gaseous product is exclusively H_2_ molecules and the soluble product is ${\text{NH}}_{4}^{+}$ ions. Based on NMR experiment, Rachiero et al. (2011b) have demonstrated that the initial product ${\text{B}(\text{OH})}_{4}^{-}$ is adsorbed over the catalyst surface without B-N adducts or intermediates desorbing. Consequently, the overall hydrolysis reaction of AB can be described as NH_3_BH_3_+3H_2_O$\overset{\text{catalyst}}{\to }$catalyst·NH_3_+B(OH)_3_+3H_2_↑, which was readily confirmed by the composition of reaction products using the x-ray diffraction analysis (Mohajeri et al., 2007; Brockman et al., 2010; Chou et al., 2012; Liu et al., 2012; Komova et al., 2016). The NH_3_ and B(OH)_3_ are being dissolved in water as described by a well known acid-base equilibrium NH_3_+H_2_O↔${\text{NH}}_{4}^{+}$+OH^−^ and B(OH)_3_+OH^−^↔${\text{B}(\text{OH})}_{4}^{-}$↔${\text{BO}}_{2}^{-}$+2H_2_O (Brockman et al., 2010; Komova et al., 2016). These final products suggest that one portion of H_2_ molecule is generated through the H-O bond breaking in H_2_O and the other is generated through the H-B bond breaking in AB, and NH_3_ molecule is formed by B-N bond cleavage. In addition, the observations of these experiments are consistent with the Langmuir-Hinshelwood mechanism for the heterogeneous catalysis (Basu et al., 2010; Rakap and Özkar, 2010), i.e., AB and H_2_O adsorb over the catalytic surface to be hydrolyzed upon the formation of the final by-product ${\text{B}(\text{OH})}_{4}^{-}$. Although great progress has been achieved in the experiments for the fast and complete H_2_ release, evaluations of the B-N bond cleavage channel and the hydrogen transfer channel have not been performed to gain a deep understanding of the kinetic route, to suggest a methodological scheme which properly describes these reactions, and to find out crucial step to tune the reactions for future application in H_2_ storage. In our opinion, this is a substantial gap in this field of research.

By using the density functional theory (DFT) calculations, herein, we investigate the reaction pathways of H_2_ release from the hydrolytic AB and examine the catalytic roles of small NiCu clusters. Experimentally, NiCu bimetallic nanoparticles have been successfully fabricated and the catalytic activity was evaluated in the hydrolytic dehydrogeneration of AB (Lu et al., 2014; Zhang et al., 2016). The results shown that NiCu bimetallic nanoparticles exhibit the excellent catalytic activity and the low activation energy barriers owning to the charge transfer between Cu and Ni atoms (Lu et al., 2014; Zhang et al., 2016). Since the dehydrogenation reaction of AB involves an ionic recombination of hydridic H^δ−^ and protic H^δ+^, the positively Ni atom would play an attractive role to interact with the [OH]^−^ group by hydrolyzing H_2_O while the negatively Cu atom would act as an agent to accept the hydrolyzed H^+^ ion. Considering the dissociation of NH_3_ molecule from AB in the experiments, we naturally deduce the hydrolytic dehydrogenation of AB that the hydridic H(B) atoms from –BH_3_ edge of AB would like to interact with the protic H(O) atoms from H_2_O, aggregating and forming three H_2_ molecules. Firstly and importantly, validation of the aforementioned rules and trends is crucial and necessary from the electronic structure. Secondly, the potential energy surfaces on the reaction channels describe the heights of activation energy barriers and thermodynamic stabilities, which can be used to compare with the experimental measurement. Finally, our results are important in developing the evidence-based experimental requirements for the safe handling of this hydride in everyday laboratory and industrial practice. In what follows, we will firstly describe the computational methodology used in the work in section 2, and then present our results and discussions in section 3. Finally, a summary is given in section 4.

## 2. Methods

Our calculations have been performed by using the Density Functional Theory (DFT) based DMol^3^ code (Delley, 2000), where the Perdew-Wang (PW91) exchange-correlation potential (Becke, 1988; Perdew and Wang, 1992) to the generalized gradient approximation (GGA) (White and Bird, 1994) was adopted. The double numerical basis sets with the *p*-polarization function (DNP), all electrons treated as the valance electrons, and the thermal corrections were employed in the calculations for the equilibrium geometries of all molecular species, i.e., the reactant-complex (R), transition states (TS), intermediates (IM), and products (P). The DNP basis set is quite a bit better than Gaussian 6-31G** basis set under the accurate level, and the former one is more effective than the latter one (Hirshfeld, 1977; Wu et al., 2013). The harmonic vibrational frequencies were determined at the same level to confirm whether the optimized structures are local minima (no imaginary frequency) or transition states (one imaginary frequency) and to evaluate the zero-point vibrational energy. To obtain the TS, linear synchronous transit (LST) and quadratic synchronous transit (QST) methods were firstly utilized to execute an extensive search, and then the TS optimization has been done with the eigenvector-following methods, following which the TS confirmation was performed to produce a refined reaction path based on LST or QST (Peng and Schlegel, 1993; Peng et al., 1996; Ayala and Schlegel, 1997). The convergence criterions were set to 2 × 10^−5^ Ha for the maximum energy change, 4×10^−3^ Ha/Å for the maximum force, 5×10^−3^ Å for the maximum atomic displacement, respectively. The activation energy barrier (E_act_) determines whether or not the reactions take place spontaneously and reach to the final state without the extra-energy absorbed from the outside. It was calculated as the energy difference between the initial reactants and the highest transition state of a whole reaction pathway, i.e., E_act_ = E_HTS_-E_React_. The energy values hereafter are enthalpy except for specially mentioned. The zero-point energy and enthalpy corrections have not been considered, because the corrections always reduce the values in a small magnitude (Zhou T. et al., 2016).

**Figure 1:**
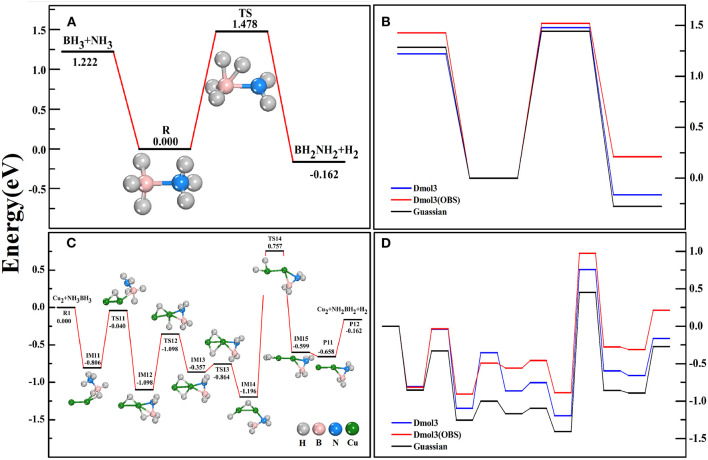
The reaction pathways of AB dehydrogenation without **(A)** and with **(C)** the catalyst Cu_2_ dimer. The comparison of counterpart reactions in Gaussian and DMol^3^ software **(B,D)**.

For free AB, we found that the bond-lengths of B-N, B-H, and N-H bonds are 1.66, 1.22, 1.02 Å, respectively, which are in good agreement with the experimental measurements (Thorne et al., 2011) and theoretical values under more precise calculations (Gaussian-B3LYP/6-311+g^*^) (Zhou T. et al., 2016). Thus, we have a high degree of confidence in our structural configurations. To validate our method in calculating the dehydrogenation dynamics, we have calculated the dehydrogenation pathways of AB without (Figures 1A,B) and with (Figures 1C,D) the help of Cu_2_ catalyst by using the DMol^3^ and Gaussian methods. From Figure 1, we noted that the DMol^3^ method (BPW91/DNP) gives a similar variational trend as the Gaussian method does (Zhou T. et al., 2016). Although the IM states and TS states under the former calculations are higher in energy than under the latter calculations, both methods report the same energy orderings of these states. Typically, the highest energy barrier (the decisive reaction step) (1.953 eV) and the exoergic energy (0.162 eV) of the whole reaction path are well reproduced by DMol^3^ method. Furthermore, the Van der Waals correction (Bucko et al., 2010) has been taken into account (BPW91/DNP/OBS) for the benchmark calculations, however, the results (red lines) shown that it does not present the superiority. Therefore, the following calculations were performed within the DMol-BPW91/DNP method.

## 3. Results

### 3.1. Synergistic Adsorption of H_2_O and AB Molecules on NiCu Dimer

**Figure 2:**
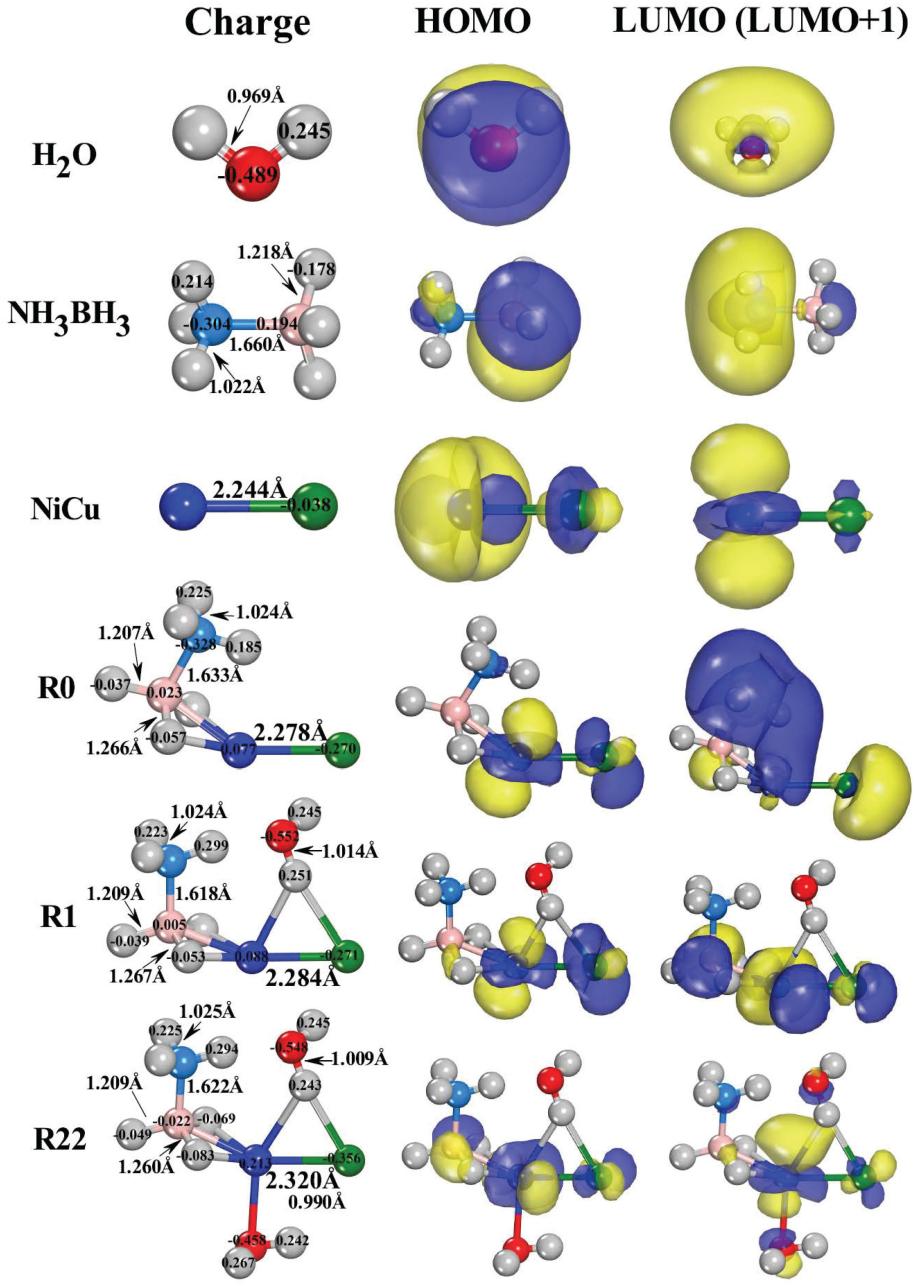
The bond-length (in Å), Mulliken population charges (in e), as well as the schematic diagram of the frontier molecular orbitals (HOMO, LUMO/LUMO+1) for the energetically favorable reactant structures.

The initial interactions between the reactants (AB and H_2_O) and catalyst (NiCu dimer) can be demonstrated by the energetically most favorable configurations shown in Figure 2. Although the AB hydrolysis reaction is complex in nature and it involves multistep mechanism, the configuration of the pre-equilibrium complex AB (reactants adsorbed on catalyst) is very important for the following reactions. By examining the possible adsorption styles of AB on NiCu dimer, including: –BH_3_ (–NH_3_) edge solely linked with Cu (Ni) atom; –BH_3_ and –NH_3_ edges synchronously linked with Cu and Ni atoms (and *viceversa*); B and N atoms directly linked with Cu (Ni) atom, we found that the configuration within –BH_3_ edge approaching Ni atom is the most stable (R0). It is similar to the structure IM31 we proposed in previous literature (Zhou T. et al., 2016). The reason that –BH_3_ edge prefers to bond with Ni atom instead of Cu atom can be understood from the Lewis base and acid reactions (Roach et al., 2009; Reber et al., 2010; Li et al., 2013). Since the overall negatively charged –BH_3_ fragment [specifically for the hydridic H(B) donors] can be regarded as the Lewis base, it would like to interact with the positively charged Ni atom that can be regarded as the Lewis acid. In Figure 2, the highest occupied molecular orbital (HOMO), the lowest unoccupied molecular orbital (LUMO), and Mulliken atomic charges are presented on each structural configuration. From the point of view of the frontier orbital theory (Li and Evans, 1995), the LUMO/LUMO+1 site serves as the Lewis acid which will accept electrons readily, while the HOMO site serves as the Lewis base which will donate electrons. Consequently, –BH_3_ edge would like to interact with Ni atom, because HOMO (LUMO) charge distribution is primarily localized around the –BH_3_ fragment of AB molecule (Ni site of NiCu dimer).

By inspecting the changes of bond-lengths between the adsorbed AB (R0) and the free AB, we found that two H-B bonds near Ni atom are elongated by 0.048 Å while one H-B bond far from Ni atom is compressed by 0.011 Å. The B-N bond is also compressed by 0.027 Å. There are the negligible changes for two of H-N bond-lengths on –NH_3_ edge whereas the other H-N bond is stretched by 0.025Å. Since Ni/Cu atoms are positively/negatively charged in NiCu dimer, they would like to present the moderate interactions with different hydrogen species because the former/latter one is the hydridic-hydrogen/protic-hydrogen acceptor. After the adsorption, the hydridic H(B) atoms donate their partial electrons to Ni atom, resulting in H(B) atoms in the less negatively charged state (−0.178 e → −0.057 e). Meanwhile, the Ni atom back donates its electrons to B and Cu atoms, resulting in B atom in the less positively charged state (0.194 e → 0.023 e) and Cu atom in the more negatively charged state (−0.038 e → −0.270 e). Note that the drawing force of Ni atom on its neighboring H(B) atoms has stretched the B-H bond-lengths. These activated H(B) atoms are then being ready for the subsequent dehydrogenation reactions owning to the formation of the agostic-bonds [B-H(B)⋯Ni] (Brookhart et al., 2007; Zins et al., 2015).

In the hydrolysis reaction process, it has been proposed that AB molecule firstly diffuses onto the catalyst surface and forms an activated intermediate. Upon the attack of a water molecule, the intermediate of AB molecule is dissociated to release H_2_ (Chandra and Xu, 2006a,b; Xu and Chandra, 2006; Lu et al., 2013). Consequently, H_2_O molecule should to adsorb on the AB-NiCu complex. Note that the adsorption of H_2_O molecules plays a negligible role on the bond-lengths and bond-angles of AB fragment (R1 and R22 in Figure 2). For one H_2_O adsorption in R1 configuration, one protic H(O) atom is bonded with both Ni and Cu atoms, stretching the corresponding H-O bond by 0.045 Å. If O atom is bonded with Ni atom, it is 0.183 eV higher in energy. For two H_2_O adsorption in R22 configuration, O atom of the second H_2_O with the lone-pair electrons will nucleophilically attack the well-defined electrophilic Ni site, because O atom tends to donate its lone-pair electrons to the LUMO and/or LUMO+1 orbitals around the Ni site (R1). Relative to O atom in free H_2_O, we found that the O atom of the O-bonded H_2_O in R22 losses 0.031 electrons (−0.489 e → −0.458 e) while the O atom of the H-bonded H_2_O acquires 0.019 electrons (−0.489 e → −0.548 e). Although the R1 and R22 configurations present the negligible differences in bond-lengths and Mulliken charges, their HOMO (LUMO) charge distributions are significantly different. Previously, the investigation has proposed that the HOMO (LUMO) distributions can be used as a fine guidance to determine the sub-reaction pathway (Li et al., 2013). Here, the HOMO (LUMO) differences between R1 (one H_2_O adsorption) and R22 (two H_2_O adsorption) suggest that the stepwise and simultaneous H_2_O attacking would give rise to different reaction pathways.

### 3.2. The Hydrolysis of AB Catalyzed by NiCu Dimer via the Stepwise H_2_O Attack

**Figure 3:**
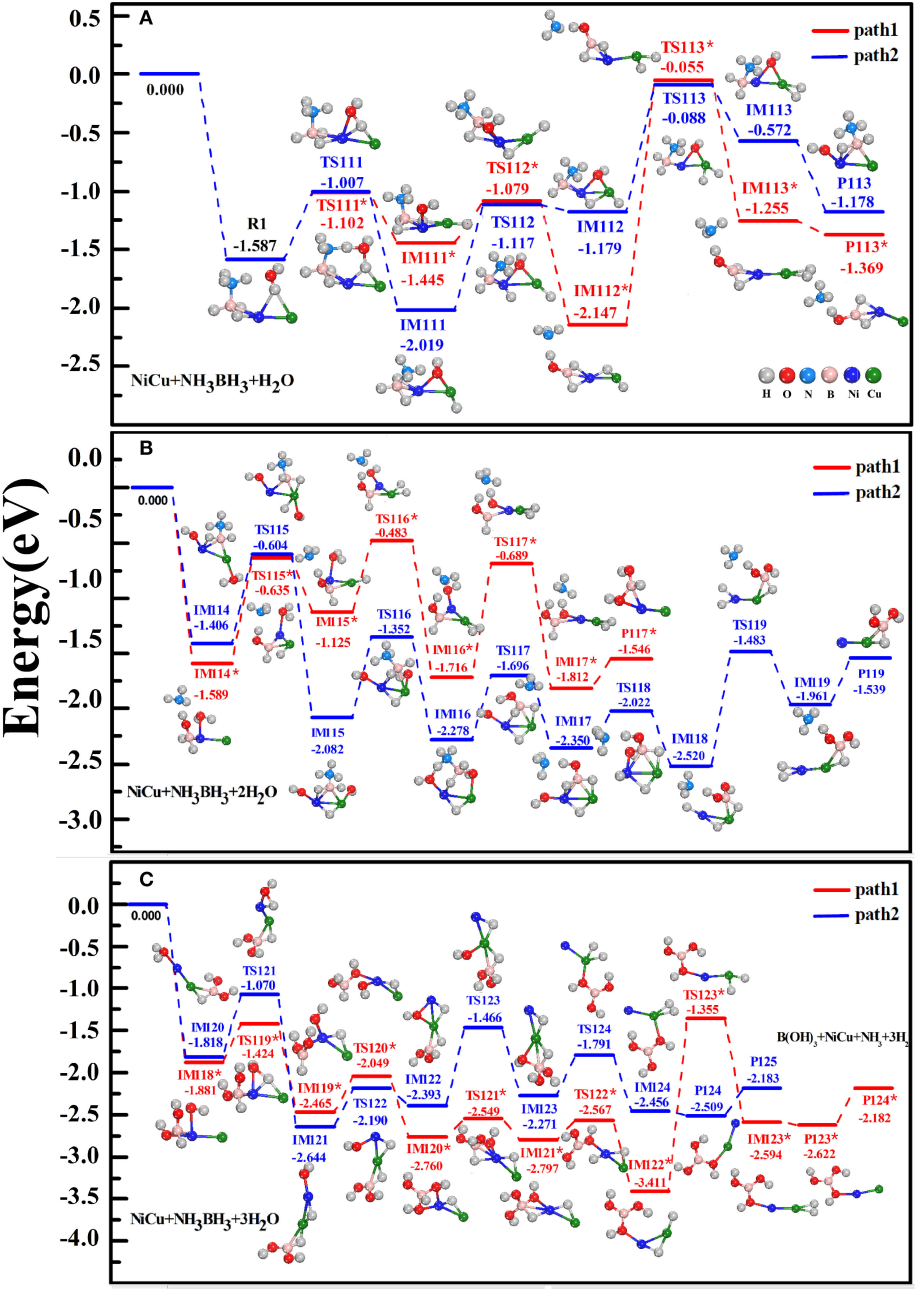
Schematic energy profiles of hydrogen production from the catalytic hydrolysis of AB in the presence of NiCu dimer. The stepwise attacks involving three H_2_O molecules, named as H_2_O^st^, H_2_O^nd^, H_2_O^rd^, are sequentially considered in **(A–C)**. The reactant, transition state, intermediate state, and product are denoted by R1, TS, IM, P, respectively.

In Figures 3A–C, we shown two preferential reaction pathways (path1 and path2) for the hydrolytic dehydrogenation of AB molecule catalyzed by NiCu dimer. These reactions undergo the stepwise attacks of H_2_O molecules, named as H_2_O^st^, H_2_O^nd^, H_2_O^rd^. The energies of R, IM, TS, P, together with their structural configurations were illustrated on each step. For the R1 reactant-complex that H_2_O^st^ and AB molecules are simultaneously adsorbed on NiCu dimer (Figure 3A), it situates at an energy level of 1.587 eV below the initial reactants AB+H_2_O^st^+NiCu. In the next reaction step, the cationic Ni atom would like to interact with the nucleophilic [–OH^st^] but the anionic Cu atom would like to interact with the protic H(O) acceptor. Under the synergistical roles of Ni and Cu atoms, the H_2_O^st^ molecule is decomposed into one hydrogen atom H(O) and one hydroxy group [–OH^st^] in IM111^*^ or IM111 state. In terms of the reaction principle for the bond activation, [–OH^st^] group will attack the positively charged B atom. If the O atom of [–OH^st^] hydroxy is close enough to the B atom, the stronger interactions between O and B atoms than between N and B atoms will split the B-N bond via the activation energy barrier of the TS112^*^ state (1.445–1.079 = 0.366 eV), i.e., –NH_3_ is dissociated from BH_3_-NH_3_ fragment in IM112^*^ state by releasing the energy of 2.147–1.079 = 1.068 eV. Previously, Banu et al. (2015) have found that when the H_2_O molecule directly interacts with BH_3_-NH_3_ fragment yet without TM catalyst, the breaking of B-N bond at the assistant of [-OH] group needs to cross a very high energy barrier of 1.19 eV, and the counterpart TS state is even 0.91 eV above the separated reactants AB+H_2_O. Clearly, the present of NiCu dimer can significantly reduce the activation energy barrier during the hydrolytic AB. If the O-B interaction doesn't play the role this time, the O atom maintains its interactions with Ni and Cu atom in IM112 state (presenting a three-membered Ni-O-Cu ring). Whichever the reaction it happens, one H(B) atom of AB is somewhat activated by Ni atom and being ready to adsorb on Cu atom, because the corresponding H-B bond in IM112^*^ is elongated by 0.169 Å (elongated by 0.056Å in IM112) with respect to 1.218 Å of free AB. Following the IM112^*^ or IM112 state, H(O) and H(B) atoms on Cu atom move close to each other to form a Cu-dihydride complex in IM113^*^ state or IM113 state. Ultimately, the H_2_^(st)^ molecule is released from the first-step-product P113^*^ ([OH]·BH_2_·NiCu+NH_3_+H_2_) or product P113 ([OH]·NiCu·NH_3_BH_2_+H_2_), which releases the energy of 0.114 or 0.606 eV.

Apart from the above dehydrogenation pathways, we have considered other reaction routes from the R1 reactants: the first dissociation of H(B) atom and then H(O) atom; the stepwise dissociations of H(N) and H(O) atoms. However, these reaction routes have not any superiority with respect to the path1 and path2. For example, the reaction that one H(B) atom in R1 is activated and interacted with Cu atom has to go across a very high energy barrier (1.146 eV). It is 0.789 eV higher in energy than IM111^*^ state. There are the possibilities that the released H_2_ molecule is comprised of H(B) and H(N) atoms from AB fragment, which is analogous to what we have proposed in Zhou T. et al. (2016). Nevertheless, our primary goal is to explore the reaction mechanism that the hydrolytic dehydrogenation is happening with the assistant of H_2_O. Consequently, these routes have been discarded owning to no dissociation of H(O) atom from H_2_O. As a mater of fact, Shevlin et al. (2011) have indicated that the dehydrogenation via the combination of the protic atoms from the N-moiety and the hydridic atoms from the B-moiety is energetically unfavored for the metal amidoboranes MNH_2_BH_3_. In addition, another possible pathway could follow the IM111^*^ or IM111 states: (i) One H(N) atom transfers to [–OH] group to produce the adsorbed H_2_O and BH_3_-NH_2_ fragment once again; (ii) One H(O) atom is dissociated from the H_2_O and then shifted to Cu atom, forming the Cu-dihydride complex and [–OH] group; (iii) The B-N bond of BH_3_-NH_2_ fragment is broken by [–OH] group, yielding the NH_2_ and H_2_ products. However, the B-N bond has an obvious contraction as one H(N) atom was migrated from –NH_3_ edge after the first step reaction. It will be of ever-increasing difficulty to break the B-N bond by [–OH] group. Typically and importantly, the released product of NH_2_ in this pathway does not agree with the experimental product of NH_3_ (Yao C. F. et al., 2008; Rachiero et al., 2011b; Chou et al., 2012; Liu et al., 2012).

For the adsorption of the second H_2_O^nd^ on the first-step-products, both Ni and Cu atoms are considered as the tentatively adsorbing sites. Our calculations demonstrate that the Ni site is 0.176 eV lower in energy than the Cu site in adsorbing O atom for the product P113^*^, whereas it reverses for the product P113. In Figure 3B, we show the subsequent reactions that the first-step-products are attacked by the H_2_O^nd^. The crucial reaction steps for the path1 are follows: (i) One of two hydridic H(B) atoms in IM114^*^ is activated by the Lewis acid of Ni atom, and then it is optimally adsorbed on Cu atom in the formation of Cu-monohydride complex in IM115^*^; (ii) The protic H(O) atom and [-OH^nd^] of the H_2_O^nd^ are synergistically activated by Cu and Ni atoms, and then this protic H(O) atom shifts to Cu atom yielding the Cu-dihydride complex in IM116^*^; (iii) The [-OH^nd^] group moves close to B atom to form the B-dihydroxy and Cu-dihydride fragments in IM117^*^, which is followed by the releasing of H_2_^(nd)^ molecule (the second-step-products P117^*^). For the successive reactions along the path2, there presents a thermodynamically downhill trend from IM114 to IM118 as well as from TS115 to TS118, revealing that these reactions would take place spontaneously due to their overall exothermic processes. In analogous with the dehydrogenation mechanism involving the H_2_O^st^ attack, three critical processes can be clearly concerned for the H_2_O^nd^ attack: (i) The protic H(O) atom is somewhat activated by Ni atom and then it links with both Ni and Cu atoms in IM115; (ii) The [-OH^nd^] group breaks the N-B bond and dissociate –NH_3_ fragment through IM116 to IM117; (iii) The B-dihydroxy and Ni-dihydride complex comes into being through IM118 to IM119, from which H_2_^(nd)^ is subsequently liberated (second-step-products P119). Generally speaking, since each energy barrier can be crossed at the available energies provided by the exothermic reaction, the overall reactions are thermodynamically feasible for either path1 or path2. Nevertheless, P119 state is the predomination state, because each IM state together with the counterpart TS state in path2 lies lower in energy than that in path1.

After the adsorption of the third H_2_O^rd^ on the second-step-products (Figure 3C), IM118^*^ and IM120 states come into being, respectively. Again, Ni atom serves as the activation site for dissociating H(O) atom from H_2_O^rd^, and its neighboring Cu atom serves as the attractive site for adsorbing H(O) atom. The last H(B) atom is gradually transferred to Cu atom to form the Cu-dihydride fragment. It is synchronously/succesively accompanied by the shifting of [-OH^rd^] group from Ni atom to B atom (forming the B-trihydroxy fragment) and by the releasing of H_2_^(rd)^ molecule. All IM and TS states are accessible for proceeding the dehydrogenation reactions. Viewing the pathway as a whole, we found that TS113^*^ and TS113 states are only 0.055 and 0.088 eV lower in energy than the initial reactants. Since they present the highest energy barriers, these states are the rate determining barriers of the overall dehydrogenation processes. Nevertheless, it is nothing to be worried about this requirement, because the foregoing reactions are overall exothermic process and TS113^*^ and TS113 states can be overcome. Ultimately, the hydrolytic dehydrogenation reaction is completed, NH_3_BH_3_+3H_2_O$\overset{\text{NiCu}}{\to }\text{NiCu}\cdot$NH_3_+B(OH)_3_+3H_2_↑, which is catalyzed by the NiCu dimer and orderly attacked by three H_2_O molecules. We can conclude that *the formation of Cu-dihydride* and *B-multihydroxy fragments in IM states is crucial for the hydrolytic dehydrogenation, and both metal atoms are acted as the catalytic sites for breaking the H-O bond of H*_2_*O and N-B bond of NH*_3_*BH*_3_.

### 3.3. The Hydrolysis of AB Catalyzed by NiCu Dimer via the Simultaneous H_2_O Attack

**Figure 4:**
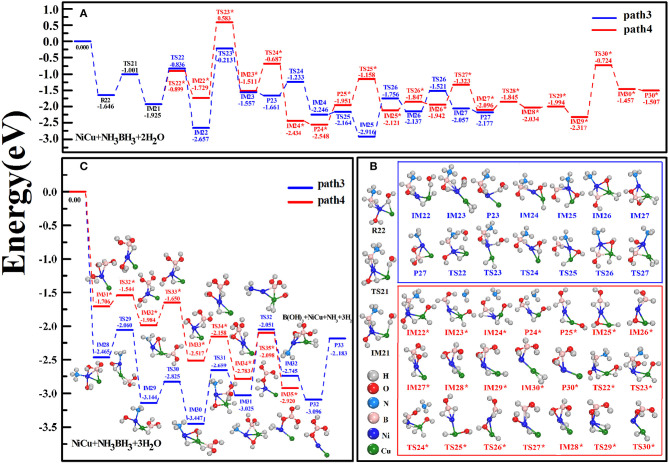
Schematic energy profiles of catalytic hydrolysis of AB involving the simultaneous H_2_O attacks **(A)**. The hydrolysis AB is simultaneously attacked by H_2_O^(1)^ and H_2_O^(2)^ **(B)** and subsequently attacked by H_2_O^(3)^ **(C)**.

For the hydrolytic AB via the simultaneous adsorption of a few H_2_O molecules, we considered the reaction case: AB is simultaneously attacked by H_2_O^(1)^ and H_2_O^(2)^ and subsequently attacked by H_2_O^(3)^. In the configuration of R22 reactant (Figure 4B), H_2_O^(1)^ with O atom is attached to Ni atom while H_2_O^(2)^ with H atom is bridged with Cu and Ni atoms. From R22 to IM22 states along the path3, one H-O bond of H_2_O^(1)^ and one H-B bond of AB are successively broken. Then, H(O) and H(B) atoms are bonded with Cu atom to generate the Cu-dihydride complex and [–OH]^(1)^ group, following which the B-monohydroxy complex (IM22) comes into being. In this situation, N-B bond-length is elongated by 0.050 Å with respect to 1.622 Å in R22. For the subsequent reaction from IM22 to IM23, H_2_O^(2)^ is shifted from Cu site to Ni site. After releasing the first H_2_^(st)^ in P23, several steps related to the atomic transfers are crucial: (i) One H(O) atom of H_2_O^(2)^ is dissociated and subsequently bonded with Cu atom (IM24), yielding [–OH]^(2)^ group that is ready to break the N-B bond of AB (IM25); (ii) One H(B) atom gradually moves to Cu atom to generate the Cu-dihydride (IM26), which is the prerequisite in releasing the second H_2_^(nd)^ (P27); (iii) The adsorption site of [–OH]^(2)^ is adjusted to connect with B atom (TS27), which contributes to the B-dihydroxy fragments (IM27). Thus, above reactions can be described as: NH_3_BH_3_+2H_2_O$\overset{\text{NiCu}}{\to }\text{NiCu}\cdot$NH_3_⋯HB(OH)_2_+2H_2_↑. When H_2_O^(3)^ is adsorbed on the resulting product P27 (Figure 4C), analogously, one H(O) atom of H_2_O^(3)^ and the last H(B) atom are gradually transferred to Cu atom (from IM29 to IM31). It is followed by the movement of [–OH]^(3)^ group to B site, again yielding the Cu-dihydride and B-trihydroxy complex (IM32). Then, the releases of NH_3_ and H_2_^(rd)^ via P32 and P33 states can be achieved. Finally, we complete the experimental reactions in an overall exothermic and barrierless pathway, NH_3_BH_3_+3H_2_O$\overset{\text{NiCu}}{\to }$NH_3_+NiCu·B(OH)_3_+3H_2_↑. On the whole, the key mechanism of the path3 is that H(O) and H(B) atoms are alternately dissociated from H_2_O and AB molecules, successively yielding Cu-dihydride and B-multihydroxy complexes in IM states.

Unlike the dehydrogenation mechanism of the path3, we consider the reaction path 4 that two H(O) atoms are firstly detached from H_2_O^(1)^ and H_2_O^(2)^ (IM22^*^) and then H(B) atoms are departed from AB molecule (IM24^*^). However, high energy barrier of 1.729–0.583 = 1.146 eV is encountered at TS23^*^ state when [–OH]^(1)^ group acts as an attacker to dissociate –NH_3_ fragment (IM23^*^). Although this reaction step is thermodynamically unfeasible, to verify our findings that the hydrolytic dehydrogenation reaction undergoes via the specific intermediate states, we continue the reactions irrespective of TS23^*^ state. To one's expectation, the formations of the Cu-dihydride complexes (IM24^*^, IM30^*^, and IM35^*^) together with the B-multihydroxy complexes (IM23^*^, IM27^*^, IM34^*^) can be clearly discerned from the sequential steps in Figures 4B,C. Experimentally, the activation energy for the catalytic hydrolysis of AB dehydrogenation has been conjectured to be the step reaction of B-N bond breaking (Xu and Chandra, 2006; Chandra and Xu, 2007), where an apparent value was estimated around 0.4 eV for CuNi catalysts (Lu et al., 2014; Zhang et al., 2016). Our E_act_ = 0.58 eV determined by the highest energy barrier of the reaction path4 at TS113^*^ state is approach to these experimental activation energies. Here, we found that the hydride transfer is responsible for the rate-determining step for all pathways we studied.

### 3.4. The Hydrolysis of AB Catalyzed by Ni_3_Cu Cluster via the Simultaneous H_2_O Attack

**Figure 5:**
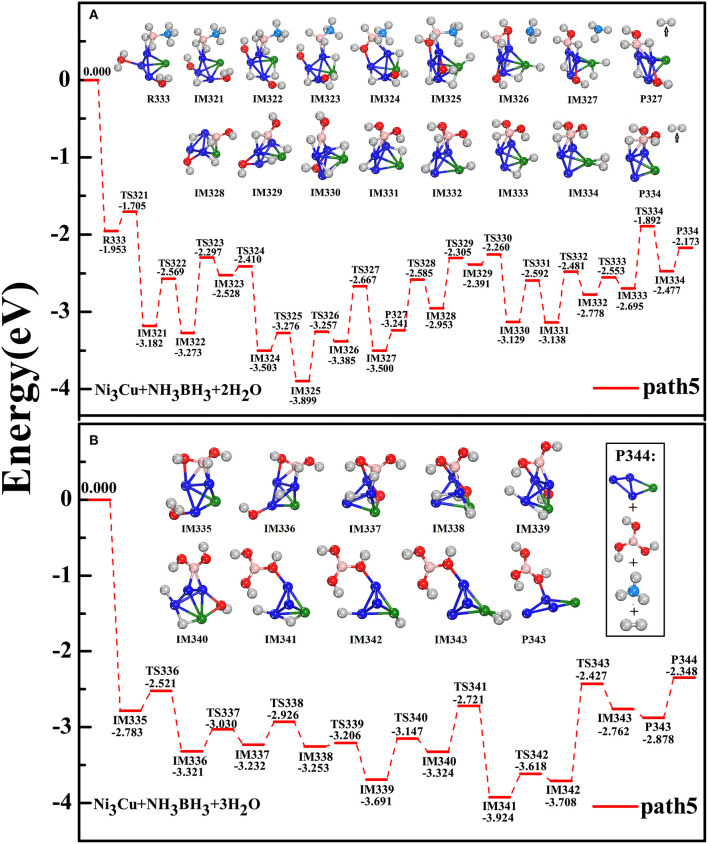
Similar to that of Figure 4 but for the catalyst of Ni_3_Cu tetramer: **(A)** hydrolysis of AB simultaneously attacked by H_2_O^(1)^ and H_2_O^(2)^; **(B)** subsequently attacked by the third H_2_O^(3)^.

Since the tetrahedral structure is the block-unit for constructing large metal nanoclusters, Ni_3_Cu tetrahedron cluster is treated as an ideal model to underline the catalysis effect on the hydrolytic dehydrogenation of AB. Importantly, experimental synthesizing has shown the Cu@Ni core-shell structure, where Ni atoms are on the structural surface and as the active sites (Lu et al., 2014; Zhang et al., 2016). After considering the different adsorption styles, i.e., -BH_3_ fragment (or -NH_3_ fragment) is adjacent to Ni atom (or Cu atom) as well as H_2_O^(1)^ and H_2_O^(2)^ molecules are attached on various metal sites, we found that R333 configuration is the most favorable adsorption style (Figure 5). Although the reaction steps are more and complex in the present of Ni_3_Cu cluster, according to the criterion of the dehydrogenation that the reactions should follow the pathway in an exothermic, overall barrierless, and the minimum energy profile, we found that the accessible pathway for the catalytic dehydrogenation by Ni_3_Cu cluster is exactly identical to that by NiCu dimer. The subsequent reaction steps are following: the alternation of the breaking of three H-O bonds from three H_2_O molecules (IM321, IM322, IM336) with the breaking of three B-H bonds from AB molecule (IM323, IM328, IM338); the dissociation of NH_3_ from AB molecule (IM326); the adjustment of the adsorbing sites of H(O) and H(B) atoms and [-OH] groups to yield the metal-dihydride and B-multioxhydryl fragment (IM327, IM334, IM343); the liberation of three H_2_ molecules through three intermediate products (P327, P334, P343); the recovery of Ni_3_Cu catalyst after the complete reactions (P344). Throughout all procedures of the path5, the hydrolytic dehydrogenation process is feasible both in kinetics and thermodynamics: NH_3_BH_3_+3H_2_O$\overset{{\text{Ni}}_{3}\text{Cu}}{\to }{\text{Ni}}_{3}\text{Cu}\cdot$NH_3_BH_3_⋯(H_2_O)_3_ → Ni_3_Cu·NH_3_+B(OH)_3_+3H_2_↑.

## 4. Conclusions

**Figure 6:**
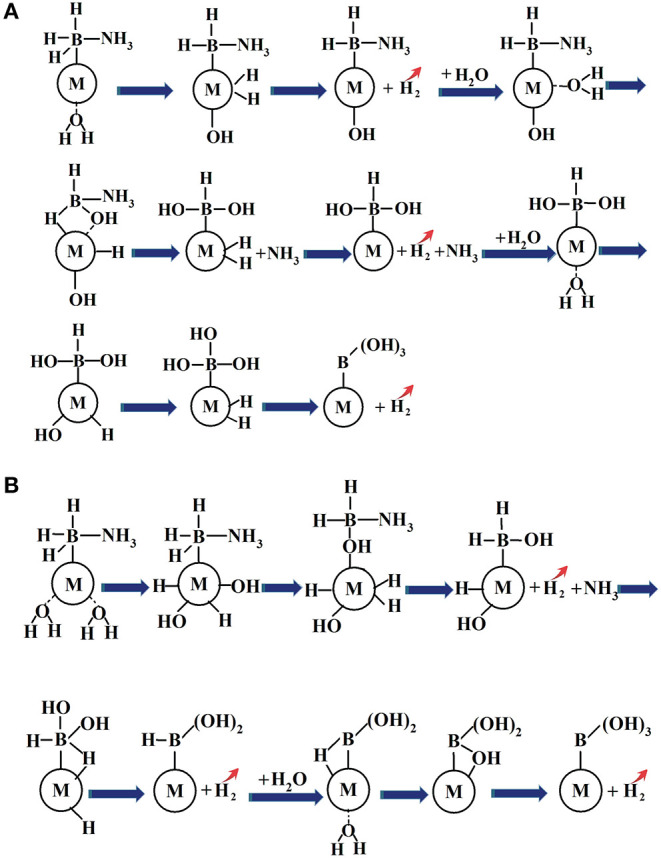
The proposed schematic representation of hydrolytic AB for hydrogen generation in the presence of metal catalyst: **(A)** hydrolysis of AB catalyzed by metal catalyst via the stepwise H_2_O attack; **(B)** hydrolysis of AB by metal catalyst via the simultaneous H_2_O attack.

A novel reaction strategy to the prediction of the dehydrogenation sequences for catalytic hydrolysis of AB by NiCu bimetal catalyst has been developed, NH_3_BH_3_+3H_2_O$\overset{\text{NiCu}}{\to }\text{NiCu}\cdot$NH_3_+B(OH)_3_+3H_2_↑, and the plausible mechanisms were analyzed under a molecular level by using the density functional theory method. Our proposed catalytic hydrolysis of AB for the hydrogen generation are illustrated in Figure 6.

The driving force behind the formations of NH_3_ and H_2_ is the development of [-OH] group and Cu-dihydride complex species after the H_2_O participation. The former resultant attack the B-N bond to dissociate NH_3_ and the latter resultant is the prerequisite toward the H_2_ elimination. The activation energy barrier for the hydrolytic AB is most likely caused by the hydride transfer from H(B) to metal atom rather than the B-N bond breaking. The formation of heterometallic NiCu bonds and the charge transfers might be the key factor to tune the frontier molecular orbitals of the catalyst surface atom, which play the decisive role to react with the molecules (AB and H_2_O) and to stabilize the possible intermediate states, leading to the improved catalytic activity and selectivity in comparison with those of the corresponding monometallic counterparts.

## Data Availability Statement

All datasets generated for this study are included in the article/supplementary material.

## Author Contributions

WY, YZ, and TZ: software and writing-original draft preparation. GW: software, writing-original draft preparation, and methodology. DW: writing-reviewing and editing. HY: software, writing-original draft preparation, methodology, writing-reviewing and editing, and conceptualization. All authors contributed to the article and approved the submitted version.

## Conflict of Interest

The authors declare that the research was conducted in the absence of any commercial or financial relationships that could be construed as a potential conflict of interest.
